# Crosstalk Between the Gut Microbiota and the Brain: An Update on Neuroimaging Findings

**DOI:** 10.3389/fneur.2019.00883

**Published:** 2019-08-13

**Authors:** Ping Liu, Guoping Peng, Ning Zhang, Baohong Wang, Benyan Luo

**Affiliations:** ^1^Department of Neurology, The First Affiliated Hospital, Zhejiang University College of Medicine, Hangzhou, China; ^2^Department of Neurology, Pujiang People's Hospital, Pujiang, China; ^3^State Key Laboratory for Diagnosis and Treatment of Infectious Diseases, Collaborative Innovation Center for Diagnosis and Treatment of Infectious Diseases, The First Affiliated Hospital, College of Medicine, Zhejiang University, Hangzhou, China

**Keywords:** gut microbiota, microbiota-gut-brain axis, neuroimaging, mutimodal, mutual communication

## Abstract

An increasing amount of evidence suggests that bidirectional communication between the gut microbiome and the central nervous system (CNS), which is also known as the microbiota-gut-brain axis, plays a key role in the development and function of the brain. For example, alterations or perturbations of the gut microbiota (GM) are associated with neurodevelopmental, neurodegenerative, and psychiatric disorders and modulation of the microbiota-gut-brain axis by probiotics, pre-biotics, and/or diet induces preventative and therapeutic effects. The current interpretation of the mechanisms underlying this relationship are mainly based on, but not limited to, parallel CNS, endocrine, and immune-related molecular pathways that interact with each other. Although many studies have revealed the peripheral aspects of this axis, there is a paucity of data on how structural and functional changes in the brain correspond with gut microbiotic states *in vivo*. However, modern neuroimaging techniques and other imaging modalities have been increasingly applied to study the structure, function, and molecular aspects of brain activity in living healthy human and patient populations, which has resulted in an increased understanding of the microbiota-gut-brain axis. The present review focuses on recent studies of healthy individuals and patients with diverse neurological disorders that employed a combination of advanced neuroimaging techniques and gut microbiome analyses. First, the technical information of these imaging modalities will be briefly described and then the included studies will provide primary evidence showing that the human GM profile is significantly associated with brain microstructure, intrinsic activities, and functional connectivity (FC) as well as cognitive function and mood.

## Introduction: The Microbiome-Gut-Brain Axis

Bidirectional interactions between the brain and gut and their relationships with a third component, the gut microbiome, have received increasing attention in recent years (1–3). Emotional and psychosocial factors can trigger gastrointestinal symptoms such as heartburn, indigestion, acid reflux, bloating, pain, constipation, and diarrhea (4). Conversely, a series of pre-clinical investigations has shown that dysbiosis and/or alterations of the gut microbiota (GM) are implicated in the pathogeneses and pathophysiologies of intestinal diseases, such as inflammatory bowel disease (IBD), as well as neurological disorders, and psychiatric conditions, including anxiety, depression, autism spectrum disorder (ASD), multiple sclerosis, Alzheimer's disease (AD), and Parkinson's disease (PD). However, clinical evidence supporting such interactions in humans remains relatively scarce (5–12).

Although a variety of mechanisms have been proposed to support interactions within the microbiome-gut-brain axis (MGBA) (13–16), the GM primarily communicates with the central nervous system (CNS) via neural, immune-related, endocrine, and metabolic signaling pathways (17). Chemically, the GM and brain communicate with each other using hormones, such as corticotrophin-releasing hormone (CRH) in the hypothalamic-pituitary-adrenal (HPA) axis, neurotransmitters, such as serotonin (5-HT), dopamine, and γ-aminobutyric acid (GABA), neuropeptides, and short-chain fatty acids (SCFAs) (14, 18–20).

Additionally, novel advanced methods have facilitated current understanding of these complex interactions *in vivo* and revealed the peripheral aspects of the MGBA. For example, 16s rRNA gene sequence analyses and/or high-throughput sequencing can demonstrate GM composition in terms of diversity and abundance (21, 22) and provide qualitative and quantitative information about bacterial species and changes (23). Likewise, biochemistric and molecular biological methods can identify metabolic, immunological, and endocrine molecules from different body fluids and tissues. Meanwhile, advanced neuroimaging methods have emerged as an effective tool for understanding the structure, function, and molecular aspects of the brain, which is the central component of the MGBA. Using such techniques, imaging parameters can also aid *in vivo* explorations of the potential associations between the microstructural patterns or functional conditions in the brain and particular dysbiotic states in the gut (24).

The present review assessed studies (see Table 1) of healthy individuals and patients with diverse neurological disorders that combined advanced neuroimaging techniques with GM analyses. First, the technical information of various neuroimaging modalities will be presented and then the published results of brain imaging and GM analyses as well as their correlations in healthy subjects and patient populations will be discussed.

**Table 1:** Crosstalk between the GM and human brain function.

**Subjects**	**Intervention**	**Measures**	**Results**				**References**
			**Neuroimaging results**	**GM results**	**Correlation**	**Other**	
Healthy women	FMPP for 4w (FMPP group, *n* = 12; non-FMPP group, *n* = 12; no intervention, *n* = 13)	Task-based fMRI and rs-fMRI; GM (fecal samples)	Emotional attention task based-fMRI: sensory brain network connection strength and decreases in insular and somatosensory cortical BOLD activity↓ in the FMPP group rs-fMRI: PAG was negatively correlated with sensory/affective regions and positively correlated with cortical regulatory regions (medial and dorsolateral prefrontal cortices) in FMPP group	No significant changes in fecal microbiota composition	Four-week intake of an FMPP by healthy women affected activity in brain regions that control the central processing of emotion and sensation	/	(25)
Obese and non-obese subjects	No intervention (20 obese and 19 non-obese subjects)	MRI; DTI; FLAIR; R2^*^; GM (fecal samples); cognitive tests	See the correlation column	16S bacterial gene pyrosequencing: fecal sample bacterial biodiversity↓ in obese men	Fecal microbiota diversity was negatively correlated with R2^*^ signals in the hypothalamus, hippocampus, and caudate nucleus. The abundance of Actinobacteria was positively associated with FA in the amygdala and thalamus but negatively correlated with the R2^*^ signal in the hypothalamus	The relative abundance of the Actinobacteria Phylum was positively associated with cognitive tests related to speed, attention, and cognitive flexibility	(26)
Elderly outpatients with and without cirrhosis	No intervention Group type 1: 39 cirrhotic and 37 non-cirrhotic patients; Group type 2: unimpaired cognition (*n* = 23), amnestic-type (*n* = 25), and amnestic/non-amnestic type (*n* = 28).	Multi-modal MRI (fMRI go/no-go task, volumetry, and MRS); inflammatory cytokines; GM (fecal samples); neuropsychological tests	No significant fMRI differences in brain volumes between cirrhotic and non-cirrhotic subjects. Amnestic/non-amnestic type: activation in the central opercular cortex, post-central gyrus, and superior parietal lobule during inhibition↑. Amnestic-type type: white matter, gray matter, and total brain volumes↓, hippocampal and left thalamic volumes↓ Cirrhotic subjects: mi/Cr and NAA/Cr ratios↓ and Glx/Cr ratio↑. Amnestic/non-amnestic type: mi/Cr and Glx/Cr ratios↓	Cirrhotic subjects: *Lactobacillales*↑ and *Synergisticeae*, and *Peptococcaceae*↓. Cognitively impaired groups: decreased *Subdoligranulum, Oscillibacte*, and *Porphyromona*daceae and *Prevotellaceae*↓ and *Bacteroides*↑ Unimpaired group: increased *Fecalibacterium* and *Butyricicoccus*	Regardless of the presence of cirrhosis, beneficial taxa (*Lactobacillales, Ruminococcaceae*, and *Lachnospiraceae*) were positively linked with cognition while pathogenic taxa (*Enterobacteriaceae*) were negatively linked with cognition	Serum levels of IL-6/endotoxin↑	(27)
Cirrhosis patients with/without prior HE	No intervention (Cirrhotic without prior HE, *n =* 62; cirrhotic with prior HE, *n* = 85; controls, *n* = 40)	MRS, DTI, Systemic inflammatory assessment, GM (fecal samples); cognitive testing	MRS: Cirrhotic patients with HE: Glx↑↑ mi↓↓; Cho↓↓; Cirrhotics: Glx↑ mi↓; DTI: Cirrhotics with HE: spherical isotropy↑, FA↓	Cirrhotic patients with HE: *Staphylococcaceae*↑, *Enterococcaceae*↑, *Porphyromonadaceae*↑, and *Lactobacillaceae*↑; autochthonous bacterial families (*Lachospiraceae*↓, *Ruminococcaeae*↓, and *Clostridiales* XIV↓	Autochthonous taxa were negatively correlated with hyperammonemia-associated astrocytic MRS changes while *Enterobacteriaceae* were positively correlated with hyperammonemia-associated astrocytic MRS changes (high Glx levels and low mi and Cho levels). *Porphyromonadaceae* were correlated with neuronal changes on DTI without being linked to ammonia	Cirrhotic patients with prior HE had significantly more advanced cirrhosis, more severe and higher levels of inflammatory markers and cognitive impairments compared to cirrhotic patients without HE	(28)
IBS patients with anxiety and depression	Probiotic: *Bifidobacterium longum* NCC3001 for 6 w (BL, *n* = 22; placebo, *n* = 22)	Task-based fMRI (fearful face backward masking paradigm); fecal microbiota; urine metabolome profiles; serum inflammatory markers; neurotransmitter and neurotrophin levels	BL reduced responses to negative emotional stimuli in multiple brain areas including the amygdala and fronto-limbic regions	No major changes in fecal microbiota composition after intervention	Reduced amygdala activity was correlated with decreases in depression scores	Depression scores↓; IBS symptoms improved; urine levels of methylamines and aromatic amino acid metabolites↓	(29)
Healthy women (*Bacteroides*-high group vs. *Prevotella*-high group)	No intervention	Emotion-induced task-based fMRI; structural MRI (DTI, T1)	*Prevotella* group: right hippocampal activity↓ when viewing negative valence images. *Bacteroides*-high group: white matter connectivity↓, cerebellum, frontal region, and hippocampal volumes↑, and nucleus accumbal volume↓	Subjects were divided into *Bacteroides*-high group and *Prevotella*-high group based on fecal microbiota analysis	/		(30)
Obese and non-obese subjects	Diet counseling (18 obese and 17 non-obese subjects)	MR relaxometry R2^*^; GM (fecal samples); Neuropsychological tests; plasma β-amyloid (1–24, 31–48) levels	MR R2^*^ relaxometry increased mainly in the pallidum, putamen, thalamus, and hippocampus in both groups over a 2-year period	A variety of gut microbiome changes in RA over a 2-year period	Shifts in *Gemmatimonadete*s, *Bacteroidetes, Proteobacteria, Caldiserica, Candidatus, Saccharibacteria, Tenericutes, Thermodesulfobacteria*, and *Chlorobi* RA were associated with increased percentages of R2^*^ in the striatum, superficial amygdala, and hippocampus. Shifts in the phyla *Fibrobacteres, Synergistetes*, and *Tenericutes* RA were reciprocally associated with right hippocampal R2^*^.	Circulating β-amyloid Ab42 levels were positively associated with changes in visuospatial constructional ability and immediate memory but negatively associated with increases in R2^*^	(49)
Healthy volunteers	Probiotic (Ecologic825, nine bacterial strains) for 4w (probiotic, *n* = 15; placebo, *n* = 15; no intervention, *n* = 15)	Task-based fMRI (emotional decision-making and emotional recognition memory); DTI; neuropsychological tests	Altered brain activation in the cingulum, precuneus, inferior parietal lobule, thalamus, and parahippocampal gyrus in the ED task and cerebellar activity in the ER task	No major changes in general fecal microbial diversity or evenness; *Bacteroides sp.↑, Alistipe sp.↑*, and the nicotinate and nicotinamide metabolic pathway of fecal microbiota↓	Probiotic ingestion improved emotional attention and memory performance, which was accompanied by changes in activity in corresponding brain regions	Self-reported behavioral measures of positive affect, cognitive reactivity, and memory performance improved	(50)
Healthy volunteers	Probiotic (Ecologic825, nine bacterial strains) for 4w (probiotic, *n* = 15; placebo, *n* = 15; no intervention, *n* = 15)	Rs-fMRI; diffusion MRI	FC in MFGN (in frontal pole and frontal medial cortex) and in DMN (in frontal lobe)↓, FC in SN (in cingulate gyrus and precuneus cortex)↑. No significant changes in structural connectivity (FA/MD)	Same as above [Bagga et al. (50)]	Probiotic intervention was -associated with -changes in FC but not structural connectivity		(51)
High-risk (HR) and ultra-high risk (UHR) subjects for schizophrenia	No intervention (high-risk group, *n =* 81; ultra-high risk group, *n =* 19; healthy controls, *n =* 69)	MRS; GM (fecal samples).	Ultra-high risk group: Cho levels in anterior cingulate cortex↑	Ultra-high risk group: at order level; *Clostridiales, Lactobacillales*, and *Bacteroidales*↑; at general level, *Prevotella* and *Lactobacillus*↑; synthesis of acetyl-CoA (belonging to SCFAs)↑	Alterations in MRS and GM function (synthesis of SCFAs) support the hypothesis that membrane dysfunction exists in schizophrenia	/	(52)

*Summary of multimodal MR imaging studies investigating the relationship between the adult GM and brain function*.

## Multimodal Neuroimaging Methods

Magnetic resonance imaging (MRI) is a widely used non-invasive technique capable of reflecting structural, functional, and metabolic brain properties. Additionally, this technique is readily translated between preclinical and clinical settings. In this section, the primary MRI sequences that have been utilized to study the relationship between the GM and the human brain will be discussed.

### Structural MRI Techniques

Typically, studies will assess gray matter structures in the brain in terms of global or regional cortical volume and thickness. With the development of novel analysis methods, several studies have demonstrated structural differences in the brain among different patient populations. For example, voxel-based morphometry (VBM) (31) allows for automated, quantitative, and objective evaluations of gray matter volume across the brain whereas diffusion tensor imaging (DTI), which is derived from diffusion-weighted imaging (DWI), is well-suited for visualizing the microstructural details of white matter *in vivo*. DTI has several metrics that quantify the degree and direction of water diffusion. Fractional anisotropy (FA), which is the most commonly assessed metric, measures the directional coherence of water diffusion within tissues and reflects the degree of structural integrity and the myelination of white matter. In addition to basic 3D visualization methods, fiber tracking has been used to delineate specific white matter tracts for quantitative analyses in various groups, including pediatric subjects, elderly subjects, and patients with schizophrenia, brain tumors, AD, or other disorders (32, 33).

Preclinical evidence has also demonstrated that the GM plays a critical role in the development and function of CNS tissues via different metabolic or immune-related signaling pathways. From a structural point of view, region-specific changes in the brains of GM-free (GF) animals have been associated with specific GM metabolites. For example, the levels of brain-derived neurotrophic factor (BDNF), which is a key regulator of synaptic plasticity and neurogenesis in the brain, are reduced in the cortex and hippocampus of GF mice (34). Additionally, synaptophysin, which is a marker of synaptogenesis, and PSD-95, which is a marker of mature excitatory synapses, are lower in the striatum of GF animals compared to specific pathogen-free animals (35). Butyrate, which is an important SCFA, is associated with the increased expression of occludin, which is a tight junction protein, in the frontal cortex and hippocampus (36). The GM is also a critical promoter of microglial maintenance in the CNS during sensitive developmental periods. Furthermore, recent increases in the specificity of neuroimaging techniques have allowed for the visualization of different tissue sub-compartments (e.g., glia vs. neurons, the soma vs. dendrites, axon diameter vs. myelin thickness or axonal density) and created the possibility of unmasking subtle microstructural changes *in vivo* (22).

Importantly, an *ex-vivo* DTI study in rats conducted by Ong (37) demonstrated that diet-specific GM populations are associated with differences in brain microstructures, particularly white matter integrity. Neuroimaging techniques have also been used to investigate the central mechanisms associated with IBS in human patients. An analysis of cortical thickness that employed structural MRI with VBM showed that female patients with IBS exhibit reduced cortical thickness in the anterior insular cortex but increased cortical thickness/gray matter volume in the post-central gyrus. However, the findings of DTI studies are inconsistent in terms of changes in FA (38).

### Functional Neuroimaging Techniques

In terms of functional changes, functional MRI (fMRI) analyses using blood oxygenation level-dependent (BOLD) signals remain one of the most widely used methods for mapping and studying the neural basis of human cognition in both healthy and dysfunctional brains (39). Conventionally, alterations in neural activities can be recorded by asking the subject to perform a task designed to target a specific cognitive process. The so-called task-based fMRI paradigm tracks task-specific patterns of activition and yields important insights into how the brain responds to external stimulation. These stimuli can be visual, auditory, or other sensory modalities depending on the desired behavioral manipulation.

It is also well-known that the human brain is operational under resting conditions or in a relaxed state. Resting-state fMRI uses the BOLD signal to measure spontaneous fluctuations in the brain in the absence of conscious mentation (i.e., the “resting state”) to reflect baseline neural activity in selected regions. This technique can also be used to construct global or local brain networks based on the identified interactions. Furthermore, analyses of functional connectivity (FC) between multiple spatially distributed brain regions have revealed different resting-state networks (functionally linked brain regions) that have specific functions and varied spatial topology. Despite the fact that different resting-state network studies have used various statistical methods (e.g., seeds, independent component analysis [ICA], or clustering), different groups of subjects, and/or diverse MR acquisition protocols (e.g., multiple vendors, multiple field strengths of 1.5T, 3.0T, or 4.0T), they have produced consistent results; this is indicative of the robust formation of functionally linked resting-state networks in the brain (40). One particular network that has received increasing attention is the so-called default mode network (DMN), which consists of functional links among the posterior cingulate cortex/precuneus, medial frontal regions, and inferior parietal regions. In contrast to the other resting-state networks, the DMN exhibits a high level of activity during rest and deactivates during the performance of cognitive tasks (41).

Functional neuroimaging has also been used to assess individuals with functional gastrointestinal disorders during gut stimulation. For example, peripheral factors such as gastrointestinal tract sensation, motility, and GM composition-associated mechanical and chemical signals (e.g., immune-related or endocrine signals) induce different functional changes in the brain that are related to the sensory processing of gut homeostatic conditions (e.g., in the brainstem sensory nuclei, thalamus, and posterior insula), emotional responses (e.g., in the locus coeruleus, amygdala, hippocampus, subgenual, and pregenual anterior cingulate cortices), and top-down modulation systems (e.g., in the periaqueductal gray [PAG], rostroventral medulla, prefrontal executive control area, and anterior midcingulate cortex) (42).

### Magnetic Resonance Spectroscopy

In human patients with brain disorders, metabolic changes often precede anatomical changes. However, magnetic resonance spectroscopy (MRS) can provide unique information about the metabolic and neurobiological substrates of the brain, including the levels of N-acetylaspartate (NAA), choline (Cho), creatine (Cr), myoinositol (mi), glutamate (Glu) + glutamine (Gln), glucose, and GABA (43). In the adult brain, NAA is found almost exclusively in neurons and serves as a marker of neuronal density and viability, and changes in Cho resonance are commonly associated with diseases that alter membrane turnover and processes that are accompanied by hypercellularity. MRS has been applied to investigate a variety of neurological and neurosurgical disorders, including neoplasms, metabolic encephalopathy (hepatic encephalopathy [HE]), mitochondrial encephalopathy, and central neurodegenerative diseases (e.g., AD and PD) as well as psychiatric disorders, such as depression (44). Interestingly, using MRS on a 7T animal MRI system, Janik et al. (45) demonstrated that oral *Lactobacillus rhamnosus* increases the levels of central Glx, total NAA (tNAA; NAA + N-acetyl-aspartyl-glutamic acid [NAAG]), and GABA over different administration time courses. Additionally, proton MRS results in patients with Crohn's disease (CD) revealed higher Glu/total Cr (tCr; Cr + phosphocreatine) levels but lower GABA+/tCr levels in CD patients with abdominal pain compared to non-pain CD patients and healthy controls (46); these findings indicate that an imbalance between Glu and GABA may play a key role in abdominal pain processing. Taken together, these studies suggest that MRS is an appropriate and non-invasive technique that can be used to track neurochemical changes consequent to alterations of the gut microbiome.

### Brain Iron Deposition Imaging

Iron is the most abundant metal in the brain and is actively involved in many fundamental biological processes, including oxygen transportation, DNA synthesis, mitochondrial respiration, myelin synthesis, and neurotransmitter synthesis and metabolism (47). Additionally, iron-mediated oxidative stress has been linked to motor system degeneration and cognitive impairments and is considered to be an important pathogenetic component of neurodegenerative diseases such as PD, AD, amyotrophic lateral sclerosis (ALS), and Huntington's disease (48, 53).

Due to the paramagnetic nature of iron, advancements in MRI techniques have opened a new window into *in vivo* iron deposition imaging of the human brain. Different MRI techniques and methods have been proposed for the visual and quantitative mapping of brain iron; these options include the visual rating of T2-weighted images, R2/R2^*^ relaxometry (R2 = 1/T2 and R^*^ = 1/R2), MR phase imaging, susceptibility-weighted imaging (SWI), and quantitative susceptibility mapping (QSM). Of these methods, R2^*^ relaxometry and QSM have a high sensitivity for iron and a linear relationship with iron deposition (54, 55); however, only R2^*^ relaxometry can distinguish calcifications from iron deposits.

It has been shown that commensal bacteria such as *Bifidobacterium longum* and *Bacteroides fragilis*, which are representative members of the GM, affect hepcidin expression, which is a central regulator of systemic iron metabolism. For example, using the SWI technique, Dong Lin demonstrated that patients with hepatitis B virus (HBV)-related cirrhosis, usually in a state of gut dysbiosis (17), exhibit decreased serum levels of hepcidin and an overload of systemic iron that are linked to the excessive accumulation of iron in the basal ganglia (56). However, there is a need for more detailed studies to fully explore the interactions between the GM, systemic iron metabolism, and brain iron deposition as well as the resulting effects in healthy human subjects.

Recently, De Santis et al. (22) proposed the novel term “radiomicrobiomics” for the combined analysis of large amounts of data (i.e., “omics”) that represent an entire set of image-based brain signatures and features of microbiota. The data in this framework could be used to generate or test hypotheses and/or develop decision support tools associated with disease biomarkers and treatment. In addition to pre-clinical studies, emerging translational studies have also investigated crosstalk between the GM and CNS in humans. Future studies should incorporate measurements or interventions of gastrointestinal microbiota with neuroimaging modalities to elucidate this relationship further.

In the abovementioned studies, the enrolled subjects were from mainly healthy populations and the patient groups did not have significant gut disorders or had gut states that were similar to the control group (e.g., IBS patients). Additionally, of these studies, five were intervention studies; four required subjects to use probiotics for 4–6 weeks (25, 29, 50, 51), and one employed diet counseling (49). The assessments of brain function were mainly in the domains of cognition and mood, and included memory, executive function, attention, speed, depression, and anxiety tests. Although various neuroimaging strategies were used, most of these studies employed multimodal MRI approaches that involved both functional and structural MRI techniques. More specifically, the fMRI methods included resting-state fMRI or emotion- or cognition-induced task-based fMRI analyses of intrinsic brain activities and FC among brain networks whereas the structural MRI methods included DTI for white matter, R2^*^ for iron deposition, and volumetric analyses. MRS was used to assess regional brain metabolism in two studies (27, 52).

Accumulating experimental evidence has shown that manipulation of the gut microbiome could modulate emotion, cognition, and/or behavior by modifying neurotransmitter levels, neuroinflammation, and brain functions (57, 58). Additionally, the administration of probiotics has been explored as a potential treatment strategy for neurological and psychiatric disorders in both experimental and clinical studies (59–62). For example, Messaoudi et al. (63) found that the consumption of a probiotic supplement *(Lactobacillus helveticus* R0052 and *Bifidobacterium longum* R0175) results in anxiolytic-like activity in rats and beneficial psychological effects in healthy human volunteers. However, corresponding human data from direct brain imaging sources remain scarce. Three studies have investigated the effects of probiotic administration on behavior, brain function, and gut microbial composition, two were in healthy volunteers (50, 51) and one was in IBS patients (29). Although there were no major changes in the general microbial diversity or evenness in the fecal samples in these studies, the administration of probiotics had definite effects on brain activity and FC that were associated with emotion and memory processing. Notably, probiotic administration is associated with the reduced engagement of an extensive brain network in response to an emotion recognition task (25) and emotional stimuli (29). Taken together, these results may provide novel approaches for the prevention and treatment of psychiatric disorders, including anxiety and depression.

In terms of brain microstructure, DTI is widely used to evaluate the integrity of white matter. Using rat models, Ong et al. (37) identified global changes in white matter structural integrity due to different diet patterns, i.e., standard/control diet, high fat diet, high fiber diet, and high protein/low carbohydrate diet. On the other hand, a study of healthy volunteers did not reveal any significant regional differences in FA or mean diffusivity (MD) after a 4-week probiotic intervention (51). Interestingly, two studies from Spain investigated the interactions between the GM, brain iron deposition (R2^*^), and cognitive performance in obese and non-obese subjects and found that GM diversity is negatively linked to R2^*^ in the hypothalamus, caudate nucleus, and hippocampus (26, 49). Moreover, these authors reported that the changes in GM composition are associated with brain iron deposition and cognitive function. For example, increases in bacteria belonging to the *Tenericutes* phylum parallel decreases in R2^*^ gain in the striatum and better visuospatial constructional ability (49). These authors speculated that the bacterial metabolization of arsenic and the generation of siderophores were the mechanisms underlying these protective associations.

Regarding neurological disorders, hepatic encephalopathy (HE) presents as a spectrum of neuropsychiatric symptoms that range from subtle fluctuations in cognition to coma (64). Alterations in the GM as well as related metabolomes, such as amino acid metabolites and endotoxins, could lead to the occurrence of HE when occurring against a background of intestinal hyperpermeability (i.e., leaky gut) and systemic inflammation. Using a combination of cognitive testing, assessments of stool microbiota, brain MRI analyses, and evaluations of systemic inflammation, Ahluwalia et al. (28) identified a robust correlation network in which autochthonous bacterial families (*Lachospiraceae, Ruminococcaeae*, and *Clostridiales XIV*) are negatively correlated with liver function and glial MRS manifestations of ammonia (high Glx levels with low mi and Cho levels) in the brain, especially in subjects with HE. The same research group assessed elderly outpatients with or without cirrhosis and found that elderly patients had an altered gut-brain axis regardless of the presence of cirrhosis, which suggests that cognitive function is influenced by alterations in the GM *per se*. In another study, MRS was used to evaluate the metabolic and neurobiological substrates of the brain, and the amnestic/non-amnestic group had a decreased mi/Cr ratio and a reduced NAA-NAAG/Cr ratio in the anterior cingulate cortex. The cognitively impaired groups had a significantly lower relative abundance of genera belonging to autochthonous and beneficial taxa (27). Additionally, several studies have performed measurements of microbial or human metabolites, serum inflammatory markers, and plasma β-amyloid [1–42; (Aβ42)] to clarify the molecular mechanisms underlying these relationships. A 2-year longitudinal study by Blasco et al. (49) revealed that increases in circulating Aβ42 levels are positively associated with *Tenericutes* and *Thermodesulfobacteria* RA as well as improvements in visuospatial constructional ability and immediate memory but negatively correlated with increases in R2^*^, which may have great significance for further understanding the pathogenesis of AD.

The studies included in the present review have several limitations. First, the numbers of subjects were relatively small in most of the studies included in this review. Further studies are needed in larger number of patients to define the real effect of these changes on outcomes. Second, the samples were non-invasively obtained via direct collections of stool, which allows for the detection of a wide range of intestinal microflora but cannot differentiate between the luminal and mucosal environments, less local microenvironments, or regional differences throughout the gut. Due to the difficulty and expense associated with obtaining local tissue specimen, studies characterizing the human mucosal-associated microbiota was limited. Samples from endoscopic mucosal biopsy can directly reflect mucosal environments, and it has been reported that the composition of luminal and mucosal-associated microbiota was different both in health and certain disease states (65, 66). In particular, Keshavarzian et al. showed the mucosal and fecal microbial community of Parkinson's disease patients was significantly different from control subjects, with the fecal samples showing more marked differences than the sigmoid mucosa (67). Thus, it is important to investigate and compare the microbiota of luminal and mucosal niche. Third, the possible influences of usual diet, drugs, and exercise were not assessed. However, most studies excluded those subjects who had the history of using probiotics, prebiotics, synbiotics, or antibiotics for at least 1 month before fecal sample collection. On the other hand, body mass index between study groups was compared in some studies (30, 49). Fourth, the phylogenetic power of the 16S rRNA gene sequencing analysis was low at the species level, which requires careful treatment, and should be replicated by future metagenomics sequencing. Metagenomics is the most recent development in the study of the gut microbiota. It can provide higher taxonomical resolution than 16S rRNA sequencing, reaching the species and strain levels. Further more, it can also characterize the function of a given community (23, 68).

## Conclusions and Future Perspectives

**Figure 1:**
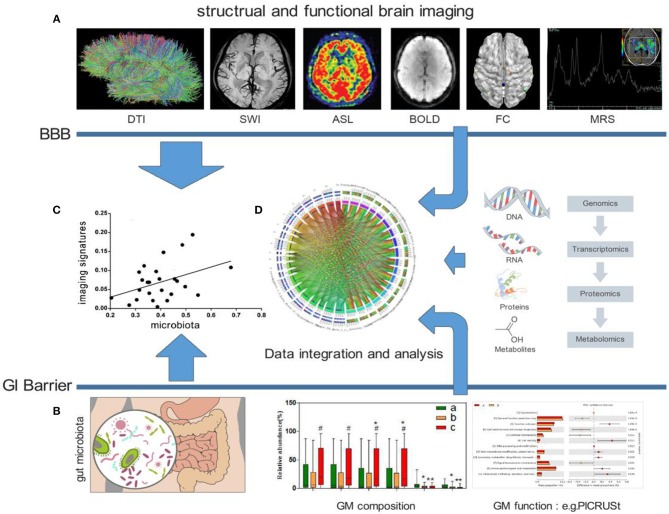
Possible frameworks for exploring crosstalk between the GM and human brain. **(A)** Examples of distinct MRI maps: DTI, SWI, ASL, BOLD, FC, and MRS (from left to right). **(B)** Analysis of the GM, including composition and function. **(C)** Explorations of the direct association between the GM and human brain imaging. **(D)** Combining other “omics” techniques, such as metabolomics and proteomics, to generate a complete picture of host and microbial pathways. DTI, Diffusion Tensor Imaging; SWI, Susceptibility Weighted Imaging; ASL, Arterial Spin Labeling; BOLD, blood oxygenation level dependent; FC, functional connection; MRS, Magnetic Resonance Spectroscopy; BBB, blood-brain barrier; GI, gastro-intestinal; GM, gut microbiota; PICRUSt, Phylogenetic Investigation of Communities by Reconstruction of Unobserved States.

Taken together, the studies included in this review indicate that the human GM profile is significantly associated with brain microstructure, intrinsic neural activities, and brain FC as well as cognitive function and mood. Well-designed longitudinal studies that include assessments of the gut microbial community structure and microbial metabolomics in conjunction with neuroimaging and behavioral testing will be required to establish directionality and causality. Furthermore, additional measures of inflammation, immune activation, neurotransmitters, neuromodulators, microbial metabolomics, and intestinal permeability, motility and visceral sensitivity will be useful for elucidating the interactions between the gut and brain. Future studies should also aim to integrate multiple “omics” techniques (69), such as metabolomics and proteomics, to generate a complete picture of host and microbial pathways (Figure 1).

## Author Contributions

PL and GP wrote the manuscript. NZ, BW, and BL helped to edit the manuscript.

### Conflict of Interest Statement

The authors declare that the research was conducted in the absence of any commercial or financial relationships that could be construed as a potential conflict of interest.
